# Empyema of the Antrum of Highmore

**Published:** 1896-07

**Authors:** Frederic C. Cobb

**Affiliations:** Boston, Mass.


### International Dental Journal.

Vol. XVII. July, 1896. No. 7.

### Original Communications.[1]

### EMPYEMA OF THE ANTRUM OF HIGHMORE.[2]

BY FREDERIC C. COBB, M.D., BOSTON, MASS.[3]

Empyema of the antrum of Highmore has been for years well known to surgeons both in this country and abroad, but was considered rare on account of a want of comprehension of its symptoms. Jourdain, Deschamps, Cooper, and Desault in the eighteenth century had described it, and Deschamps had even advised catheterization of the antrum by the normal orifice, a piece of advice, however, much more easily given than followed. The antrum was already opened through the canine fossa by Lamourier and Desault, while the alveolar process had been used as a point of approach by Meibomius and Cooper. These cases were of the acute and violent type, and were rare as they are at the present time. In the records of the Massachusetts General Hospital for the last twenty years I can find but about a dozen cases, and they are all of much the same character. The patient usually entered the hospital complaining of great pain and soreness over the antrum. On examination the whole side of the face was found to be red, swollen, and tender, and perhaps fluctuating at some point below the orbit. The question

[1] The editor and publishers are not responsible for the views of authors of papers published in this department, nor for any claim to novelty, or otherwise, that may be made by them. No papers will be received for this department that have appeared in any other journal published in the country.

[2] Read before the American Academy of Dental Science, March 4, 1896.

[3] Assistant Physician, Throat Department Massachusetts General Hospital; Physician to Throat Department, Boston Dispensary.

at first arose as to whether the inflammation were in the antrum or external to it and in the soft tissues only. Carious teeth were now looked for and extracted when found, and the antrum was washed, the fluid entering at the sinus and passing out of the alveolar opening thus made. The antrum was then packed, or perhaps curetted, and the patient allowed to leave the hospital in a week or ten days, with directions to wash the cavity daily through the alveolar opening. Unfortunately most of these cases occurred so long ago that it is hopeless now to follow their history. No mention of any nasal examination is recorded in any of the older ones, but in most of them carious teeth play an important part. No allusion is made to a possible specific cause, although in the light of our subsequent cases it should, I think, always be considered. Several of these cases occurred in young children from five to seven years of age, and show that where rhinitis persists in childhood antral disease should be thought of. All these cases were, of course, of a violent type and usually not difficult of diagnosis. It is only, however, in the last ten years that the attention of laryngologists has been drawn to a milder form of the same affection. Within the last few years much has been written about the antrum and other sinuses, and light has been thrown upon many obstinate cases of rhinitis which were formally considered incurable. When we read the reports of the cases I have mentioned it is hard to realize that the disease, as we meet it in laryngological and in surgical clinics, is the same, and that its different symptoms are simply due to the occlusion or patency of the antral orifice. These milder cases we may call latent empyema, or blennorrhœa of the antrum, and instead of the violent symptoms complained of in the form of which I have spoken, are usually signalized by a discharge of a more or less purulent character from one or other nostril, usually accompanied by a foul smell, unlike the smell of ozæna, to the patient himself. This discharge may, of course, be bilateral if both cavities are involved, but is not usually so. With this history there arises a consideration of the conditions which may give rise to a unilateral nasal discharge, and these are a foreign body in the nostril, syphilitic disease, and empyema of one of the accessory cavities. On examination of the nose we find the passage clear, thus eliminating foreign bodies from our list. If there is no sign of syphilis, no necrosis of the septum or perforation of its bony wall, we can rule out nasal syphilis with probability. We are then left with an empyema of a sinus, and must make a careful diagnosis as to which accessory cavity is affected. We have to consider in the order of frequency antral, frontal, ethmoid, and sphenoid empyema. Disease of the antrum is by far the most common, probably on account of the connection of that cavity with the teeth, many authors believing that it is ten times as frequent as empyema of any other sinus. Of course, the first point to be looked for is the location of the discharge. If pus is seen issuing from under the middle turbinate, the diagnosis is simplified, since the frontal sinus, the anterior ethmoidal, and the antrum have their outlets in that region. The nostril should therefore be carefully cleansed and wiped dry with a pledget of cotton, and then the point of issue of the pus carefully noted. If the pus comes from under the middle turbinate, we next have to differentiate between the antrum, the frontal, and anterior ethmoidal cells. This is often a difficult matter, and many expedients have been resorted to in deciding which cavity is affected.

The presence of necrosed bone in the ethmoid region and the location of the pain may determine the diagnosis in favor of that cavity. If these signs are wanting, we are left to make a diagnosis between frontal and antral empyema. The location of the pain is here not absolutely reliable, for frontal pain is met with in empyema of the antrum. We may find redness or tenderness over the frontal sinus, and it is recommended to probe the sinus through its opening in the infundibulum. If this fails the anatomical exits of the sinuses may be taken advantage of for the purpose of diagnosis in the following way: As the frontal and ethmoidal have their openings at their lowest parts, while the antral opening is at its highest part, it is evident that the two first sinuses are best drained when the head is in an upright position, but the antrum when the head is inverted. The patient is therefore directed, after his nostrils have been wiped clear of pus, to put his head between his knees, and if, after a few moments, on assuming the erect position, the nostril is found full of pus, it has probably come from the antrum. The method of transillumination by putting a small electric lamp in the mouth and darkening the room is sometimes useful, as is shown in the cases to-night. Transillumination, however, does not always give certain results, for the variation in the thickness of the walls of the antrum may be considerable, and the skin may be too thick to transmit light well. If these methods fail recourse must be had to exploratory puncture by the lower meatus, canine fossa, or alveolar process. The former is preferable, in my opinion, for the following reasons: First, the wall is usually very thin, and, second, there is little or no reactionary inflammation. I have found, in perforating through the canine fossa, an immediate swelling of the cheek, which much distorted the face for some days. The passage of the canula was painful and seemed to increase the swelling. The alveolus is often thick and therefore more difficult to use as a means of approach. It is, however, on account of its connection with the lowest part of the cavity, and on account of enabling the patient to wash out his own antrum, rapidly becoming the favorite location for puncture. In two of my cases, after an exploratory opening had been made in the lower meatus, carious teeth having their roots in the antrum were extracted and the cavity washed through their sockets, but in both cases the patient preferred to have the washing continued by the lower meatus. If it is decided to puncture by the lower meatus a strong trocar should be introduced below the lower turbinate and turned obliquely outward as far as the nostril will allow, and then pushed through into the sinus. Often, however, the bone is too thick to permit of this proceeding; it is therefore better, I think, to use a trephine or bur propelled by a dental engine. When the trephine has entered, this is at once felt by the sudden lack of resistance, and it is withdrawn, a canula of the same size fitted to an aspirating syringe introduced, and some of the contents of the antrum removed. For accurate diagnosis this proceeding is better than at once washing out the pus, because the fluid passing over the middle meatus may carry with it pus from the infundibulum, which has not necessarily come from the maxillary sinus. Usually the first washing is accompanied by a very considerable discharge of intensely foul-smelling pus. Ziem has, however, reported very slight amounts of pus from apparently plain cases of empyema, and I have noticed, in two of the cases reported to-night, so little discharge on puncture that the diagnosis seemed to me at the time uncertain, although the result of treatment appears to me proof positive, for the foul discharge improved after the first washing of the antrum and ceased in a short time, although it had lasted many weeks before the puncture. If the pus be too thick to be drawn through an aspirating tube, washing is in reality the only method of ascertaining its presence. In such cases the nostril should be carefully wiped out before washing the antrum. The odor from pus in long-standing antral empyema is almost unbearable, and one can easily understand the distress its constant presence in the nose must cause the patient. Frequently in suspected cases at the time of observation no pus is seen in the middle meatus and the surgeon is obliged to make the patient lower his head in the manner already described, so as to drain the antrum. Often the patient's history—that pus appears when he lies on the sound side—will give the observer a hint as to the best way of obtaining the discharge. I have found a peculiar thin brown mark on the handkerchiefs, a valuable guide to diagnosis which I have not seen described in papers on the subject. This stain has disappeared in the cases treated and cured, and much lessened in those constantly washed. In one case, where nothing could be seen in the nostril and yet a constant thin brown spot on the handkerchiefs appeared, a probe wound with cotton was inserted under the middle turbinate, and on being removed the stain on the cotton and on the handkerchiefs was found to correspond very well. The antrum was opened through a tooth-socket and washed and cured, the stains disappearing from the handkerchief after a few washings.

The opening was allowed to close eight months ago, and there has been no recurrence of discharge or stains since. Mackenzie has suggested that the pus discharge be examined for bacilli, and this has been done, with as yet no important results as regards diagnosis. The staphylococci pyogenes aureus and albus and citreus, and the pneumococcus of Telamon-Fränkel, have been found, the latter of interest since pneumonia is recorded as having followed antral disease. The prognosis in cases as regards time of continuance is not of the best, an untreated suppuration lasting many years or through life. It may give rise to many complications directly due to an affection of its neighboring organs or structures, and indirectly to disease of distant organs. Of the neighboring organs the eye is perhaps most frequently affected, and we have iritis, panophthalmitis, narrowing of the field of vision, orbital abscess, and lid abscess, all following empyema of the antrum. Of the skin of the face, facial abscess and pyodermatoses are mentioned, while in ear and throat acute otitis media (from entrance of pus into the Eustachian tube) and peritonsillar abscess (probably from the passage of pus over the tonsillar region) have been noted. In more distant organs we find reported pneumonia, lung abscess, arthritis, and nephritis. In none of the cases shown by me have any of these complications occurred. As to the question of treatment of empyema of the antrum there is much diversity of opinion. In the fulminant cases there is no question that a wide opening and drainage are imperative. In latent cases, on the other hand, the results of brilliant surgery do not seem to be as gratifying as in some other localities. It goes without saying that the cause must be carefully sought if we are to cure the condition. The most common causes of empyema of this sinus are, I think, in the order of frequency, carious teeth, nasal obstruction, and syphilis. Other less frequent agents of suppuration are foreign bodies in the antrum, such as supernumerary teeth or cotton pledgets introduced into tooth-sockets by the dentist, from thence escaping into the antrum. A rubber drainage-tube has been found as an exciting cause, and had remained some years in the antrum before its discovery and removal. Epidemic influenza seems to have played a prominent part in many cases. In most of my patients the teeth have been an important factor, and in two of them antisyphilitic treatment has decided the diagnosis. I think we may say at present that empyema of the antrum is an obstinate disease, and requires the greatest persistence on the part of both physician and patient. There is, of course, a limit where patience ceases to be a virtue, and mild methods must give place to more energetic surgery, but the fixing of this limit must be dictated by personal experience. Some of my cases were cured in a short time,—two or three weeks,—some after several months, and others are still under treatment and relapse when the washing is intermitted.

With regard to the location of antral puncture authorities differ, although the weight of opinion seems to be in favor of the alveolar opening. Most of my cases have been washed through the lower meatus, although some have been washed through the canine fossa and the alveolar process. The advisability of a large or small opening into the antrum is still in dispute. Most surgeons believe in the former, while Ziem, with his enormous number of cases, advocates the latter. He, however, makes up for the smallness of the opening by using a powerful pump, which sweeps the antrum out under pressure as it were. Bosworth, on the other hand, advises making an opening in the alveolar process large enough to admit the little finger and thoroughly exploring the antrum. This certainly seems a most rational method of procedure.

Case I. Empyema of the Antrum.—Mrs. Jessie B. Family history negative. Has seven children, all living and all well. Has had no diseases except dyspepsia. Seven years ago she had a yellow discharge from the nose consequent on a confinement, the only abnormality of which was the bursting of a vein (in the leg). The nasal discharge from the first was yellow and thin. She has been subject to headaches on the same side of the head. The headaches and discharge have persisted ever since. Examination shows pus issuing from the region of the middle turbinate. Examination with the probe shows the presence of necrosed bone in the middle turbinate. Transillumination with the lamp in the mouth shows the antrum on the right side bright, while that on the left side is opaque. Therefore a diagnosis of antral empyema on the left side was made. As to causation nothing definite could be learned. There was no history of specific trouble, nor of anything pointing to tuberculosis. There were no carious teeth. With a trephine an opening was made into the antrum by the lower meatus, and a copious discharge of pus was the result. The pus was very thick, heavy, and of a very foul odor. A straight canula was introduced, and the antrum washed out every morning by Dr. Chenery, into whose service she came. The operation was accompanied with but little pain, and was not followed by any discomfort whatever. On July 18 she reported herself better than for years as regarded the discharge. The wound below the lower turbinate was covered with a small membrane, as is usual in such cases, but otherwise the nose was unaffected. The antrum was washed out for some time, but approaching confinement interrupted the treatment, and she passed out of observation.

Case II.—William C., age seventy-three, came to me complaining of an offensive discharge from the right nostril and pain on the same side over the antrum. He gave the following history: Three years ago he had a toothache on that side, and the dentist extracted the second upper molar on the right side. Soon after this he had neuralgia on and about the antrum of the same side. Subsequent to this he had no neuralgia until the ten days preceding his visit to me. On examination he had an intermittent discharge of pus from the right nostril of an offensive character perceptible to himself. His pain was severe, and the discharge very annoying. Examination showed no pus in the region of the middle turbinate, but a probe wound with cotton and passed up under it was stained a yellow-brown, like the stains on his handkerchiefs. Transillumination was negative. The question now arose as to the soundness of the first molar on the affected side. The dentist to whom the patient was sent decided that the tooth was not the occasion of the antral disturbance; the patient was not operated on therefore for another week or more; but as the discharge still continued and the pain persisted, the tooth was extracted, its roots being found to be in bad condition after extraction. The socket was washed out, and the canula introduced into the antrum a day later, with only a slight discharge of pus.

The patient's general condition being very poor, he was advised to go South on a sea-voyage on March 27. He returned on April 15, stating that the discharge had been increased by the damp air and that the pain was great. The pain was not present in the night, but came on in the morning and lasted all day, to subside in the evening. There was a slight amount of swelling over the antral region, but otherwise the previous examination was unchanged. After a second consultation with the dentist, it was decided to bore up through the tooth-socket, which had healed up in the patient's absence on his vacation. After the tooth-socket was reopened by Dr. Hamilton, the antrum was regularly washed out, at first once a day, and subsequently every second day. From the first the offensive discharge ceased, but not so the pain, which, however, was greatly lessened. On damp days the pain was at its worst, but usually yielded to clearing the antrum out with antiseptic solutions. On April 30 he was almost free from pain, but the plate which had been put in to keep the opening clear annoyed him. The intervals of washing were lengthened, but as the wash-water still contained pus, it was not deemed safe to remove the plate until July 22, when the water washings came away for some days perfectly clear. The result was awaited with some anxiety, but there was no return of the nasal discharge, although there was slight pain on damp days. Of the antiseptics used, peroxide of hydrogen yielded the best results in a weak solution at first, but afterwards in the fifteen-volume solution. It was always followed by an alkaline solution. The patient has been heard from within a few days, and has no pus discharge, but the pain, on damp days, occasionally appears. I believe this to be a neuralgia of the infra-orbital nerve, as it is not associated with any antral symptoms.

Case III.—H. C. P., aged forty-one, entered the Massachusetts General Hospital November 21, 1894, complaining of a yellow discharge from the left nostril. The discharge is said to be thick, yellow, and foul. There was headache over the frontal region, but only in the afternoons. He gave the following history: Three months ago he had a "cold" in both nostrils, accompanied with a thick purulent discharge on each side. This affected both eyes, making them red and watery. This discharge had lasted for two months. Before entrance the discharge on the right side had subsided somewhat, but still persisted. At no time did he feel any symptoms of pressure, or anything like it. On examination, a large granulating middle turbinate was seen, with pus oozing from under it. The nostrils were otherwise normal. This, of course, suggested an empyema of the antrum, and further investigation of the teeth showed an open socket of the second molar tooth, the tooth having been extracted months before. With an antiseptic probe the socket was explored, and it was found to connect with the antrum. The antrum was at once washed out, and the relief was immediate. This treatment was continued every day, and at the end of a few weeks the patient learned to do this himself, and, when seen some months later, stated that the opening had nearly closed up, and, although he washed it out occasionally to keep it clean, gave him no trouble. This case was interesting from an etiological standpoint, as it apparently started from the cold, being bilateral at first. It may seem that the bilateral discharge was due simply to a rhinitis, but the time is rather long for a simple inflammation. The second point of interest is the condition of the middle turbinate. The amount of granulation tissue was so large and exuberant as to suggest malignant disease, and the rapid subsidence under washing of the antrum was very marked.

Case IV.—Nellie M., aged twenty-one. Patient entered the hospital October 14, 1895, complaining of an offensive discharge from the right nostril. This was especially bad in the morning, but more or less disagreeable during the day. She gave the following history: Two months ago, after a severe cold, she had pain over the antral region of such severity that it kept her awake for three successive nights. A sudden and copious discharge of pus was followed by relief of pain, although the soreness and a slight swelling persisted until she came to the hospital. There were no other symptoms of importance except, perhaps, a paræsthesia of the throat, acid or highly-flavored food distressing her. Examination showed a normal condition of the nostrils, except that from under the middle turbinate of the right side oozed a small stream of pus. This and the symptoms preceding the examination made disease of an accessory cavity probable, and the question arose as to which one was affected. Transillumination showed the antrum dark on the right side, while on the left it was brilliantly illuminated. An examination of the outside of the antrum showed some slight tenderness and swelling. There was no pain on pressure over the frontal region. The teeth were next examined and found to be perfectly sound, so that it was decided to make an opening into the antrum through the canine fossa. This was done, on October 5, with a small trephine run by an electric motor. The antrum was washed out, a moderate amount of thin pus appearing in the wash-water; but, owing to the inexperience of the patient, the first wash-water was swallowed, the head being held too far back, so that much may have been lost. The next day the patient felt much better; the discharge was less offensive. The face had swollen somewhat during the operation, as is common, and in my opinion an objection to the canine fossa opening, and on the following day the disfigurement was very obvious. Passing the canula into the antrum was very disagreeable. The antrum was washed out with an alkaline antiseptic solution until November 9, when the opening was allowed to heal, as all the symptoms were very much improved, and the passage of the canula was very painful. Two days later she returned, complaining of a slight increase in discharge, which subsided rapidly, and on November 25 she had no discharge. On December 22 there was no discharge, or symptoms of any kind, and they have not since recurred.

The lessons taught by this case are that opening by the canine fossa is an unsatisfactory procedure, since the swelling of the soft tissues, which is apt to follow any trauma in the cheek, is very disfiguring. The opening is not easier to get into, it seems to me, and the pain to the patient is considerable. It also tends to show that an antrum which has lasted but a short time requires a comparatively short time to heal when washed out and kept clean. April 8, patient has been seen, and has had no return of symptoms of any kind.

Case V.—Christina D. Patient came to hospital October 27, 1895, complaining of a foul discharge from the left nostril. She gave the following history: Eight years ago her teeth were much decayed, and they were removed by an unskilful dentist, who left many roots in the gums, over which a false set of teeth was placed. A year ago she had pain and tenderness over the left antrum, with swelling in the canine fossa. About the same time pus began to flow from the left nostril, staining four or five handkerchiefs a day at the time she entered the hospital. The throat and left nostril were normal, but pus was seen issuing from the right nostril in the region of the middle turbinate. Transillumination showed darkness of left antrum, while the right side was brilliantly illuminated, and there was marked tenderness over the canine fossa. She was sent to the dental infirmary, where the roots of the teeth on the left side were extracted by Dr. Paul. No discharge of pus, however, followed the extraction of the teeth. On the 30th of October an opening was made through the left nostril with the trephine and electric motor, and a quantity of the most foul-smelling pus evacuated. The next day the improvement in the symptoms was marked. Instead of four or five handerchiefs, she had but two, and the character of the pus discharged was much less offensive. The patient was very anæmic, and iron, arsenic, and strychnine were prescribed. On the 4th of November the pain had quite subsided, and a much smaller amount of pus was evacuated. On the 11th of November peroxide of hydrogen, diluted one part to three of water, was used, followed by an alkaline wash. This resulted in a decided improvement, and on November 27 very little pus was found on washing. On December 3, on lowering the head, a few drops of pus were found in the nostrils, and two days later no pus was found on washing out the nose. The patient came at first every day, and, as the discharge improved, at less frequent intervals. On December 12 the patient had so little trouble that she stayed away, contrary to advice, until December 26, when the opening into the antrum was found to have healed. A return of the symptoms necessitated another puncture and renewed washing. She is still under treatment.

Case VI.—Constance B. Family history negative. Patient entered the hospital in October, 1895, complaining of great pain over the antrum, but had no discharge. She gave the following history: The first time she had any trouble was in May; then the teeth of the lower jaw were inflamed, and she could not easily open her mouth. She had had no nasal trouble, or any specific history. She had been admitted to the hospital in August with a swelling over the antrum, but no discharge. While here she received thirty grains of iodide of potassium, and in three weeks was discharged with entire subsidence of symptoms. Less than a month after leaving the hospital she noticed a swelling and pain over the antrum, and resorted again to iodide, but with no relief. At last, some months later, she went to a dentist, who extracted a molar and washed out the sinus, finding pus, but only temporarily relieving the pain. Before entrance the eye had begun to protrude. The head was sensitive to the touch on the scalp, and the headaches were worse at night. Examination of the nostrils was negative, except for some enlargement of the middle turbinate on the left side, but no pus was seen oozing from under it. The socket of the second molar was open, but on passing a probe into the antrum no pus was discovered, although the antrum appeared full of some rather soft tissue. Transillumination of the antrum showed a marked darkness on the left side. The remaining teeth were healthy. The question now arose, what was the cause of the intense pain? An empyema simply it was not, for the probe failed to find pus, and relief of the pain did not follow the washing out of the cavity. The diagnosis lay, therefore, between syphilitic and malignant disease of the antrum. In favor of the latter was the failure of iodide to act after the patient left the hospital, and a progressive loss of flesh and strength. In favor of the former was the subsidence of the symptoms in the hospital under specific treatment, the tenderness of the scalp, the headaches worse at night, and the absence of hemorrhage on probing. Dr. Warren made an exploratory opening, and found necrosed bones without any signs of sarcoma. The necrosed bone was removed as far as was possible, and the pain at once ceased. She was given iodide, and discharged, doing well.

CASE VII.—Maurice K., aged seventeen, clerk, entered the hospital December 4, 1895, complaining of a discharge of three weeks' duration from the right nostril, excoriating the right side of the upper lip. There was no pain and no odor and no constitutional symptoms. The right side showed darker than the left on trans-illumination. There was some atrophy of the turbinate, but no tenderness over the antrum or malar process. The diagnosis was made by an exploratory puncture, and only a small amount of pus removed. The antrum was washed several times, and on December 9 the discharge from the nose had ceased and the patient felt much better. The excoriation of the lip was nearly healed. On the 10th there was no more discharge and the treatment was discontinued. After a few days he was discharged, with orders to report if the discharge should reappear, but has not since been heard from. It seems to me that this was an acute catarrh of the antrum, as shown by the one-sided discharge and excoriation of the lip under that nostril. Its duration had been so short that it yielded readily to treatment.

CASE VIII.—Lizzie M. Patient has had considerable trouble with the teeth of the upper jaw for several years. The last three molars were removed some time ago, and the second bicuspid is at present necrotic and painful. This symptom occurred over a year ago and has persisted since. With the nose she has no trouble. Two weeks she had a sharp pain over the antrum, accompanied with fever and chills, with pain in the back and limbs. These acute symptoms lasted about a week, and were relieved by a purulent discharge from the left nostril about ten days before entrance. Examination showed pain over the antrum and a discharge coming from under the middle turbinate. The second bicuspid was carious as I have stated. An exploratory puncture was made under the lower turbinate with the trephine, and foul-smelling pus evacuated. The patient is still under treatment by washing with antiseptics, and has gained several pounds in weight and very much in general health. The discharge has not ceased, although it has much improved. Besides washing out the antrum the patient has had the carious tooth removed, and the socket was found to connect with the antrum. The interesting points about this case so far are the rapid improvement under antral washing and the fact that the bicuspid tooth connected with the antrum,—a fact not very common. So severe was the apparent cachexia that I had a small polyp, found in the middle meatus, examined microscopically in order to bar out the diagnosis of sarcoma. This growth was found to be benignant. This patient has gained ten pounds, but treatment is still necessary.

*June 20.*—Patient's discharge has entirely ceased.

Case IX.—C. M. D., aged twenty-two, entered the hospital complaining of a foul discharge from the left nostril. Patient has had trouble with his teeth on that side for two or three years, and the left second molar had been especially tender for two weeks before the onset of his symptoms. With his nose he had hitherto had no trouble. He had had no trouble of a specific nature, nor any family history of tuberculosis or new growths. Five days before entrance his acute symptoms began with sharp pain over the antrum, and severe headache and chills. The headache was over both temples and accompanied with high fever. At this time there was no discharge from the nose. These symptoms subsided as soon as the left nostril began to discharge a foul-smelling, thin secretion. The nasal discharge stained his handkerchiefs a brownish-yellow color, and was copious enough to necessitate the use of four or five handkerchiefs a day. He had still some pain in the temporal region and soreness over the antrum. Examination showed the left upper third molar to contain a large cavity, but otherwise the teeth appeared to be sound. The nostril showed pus in the middle meatus both under and over the middle turbinate. The lower turbinate was pushed over towards the septum, making the breathing space narrower than normal. There were no lesions of the skin to be seen. Transillumination of the antrum showed a marked difference in the light transmission of the two sides, for the left antrum does not transmit light as the right side does. Puncture through the lower meatus gave rise to a discharge of thin and very foul pus. The discharge after the first washing began to improve, but the pain persisted for a few days. He was washed at first every day, and later, as the symptoms improved, every second day. At present the discharge is slight and the wash-water comes away almost clear. He has been washed with peroxide, followed by Seiler's solution, but this has been given up as possibly too irritating. He seems to have received the greatest relief from injections of iodoform in solution in glycerin. So far I have not considered it wise to stop the washing on account of the well-known liability to recurrence.

*March 16.*—He has had no washing for two weeks, but I have inserted the canula to keep the opening patent. He has now no pus discharge and considers himself well.

*April 8.*—Patient has been seen to-day, and has no discharge or pain since March 2, and has not had his antrum washed or treated since that date.

*June 15.*—Patient is still perfectly well.

